# Oncology distress screening within predominately Black Veterans: Outcomes on supportive care utilization, hospitalizations, and mortality

**DOI:** 10.1002/cam4.5560

**Published:** 2022-12-27

**Authors:** Desiree R. Azizoddin, Matthew Allsop, Subrina Farah, Farah Salim, Joshua Hauser, Ashton R. Baltazar, Robert Molokie, Jane Weber, Christine Weldon, Lawrence Feldman, Joanna L. Martin

**Affiliations:** ^1^ Health Promotion Research Center, Stephenson Cancer Center University of Oklahoma Health Sciences Center Oklahoma City Oklahoma USA; ^2^ Department of Psychosocial Oncology and Palliative Care Dana‐Farber Cancer Institute Boston Massachusetts USA; ^3^ Academic Unit of Palliative Care Leeds Institute of Health Sciences, University of Leeds Leeds UK; ^4^ Center for Clinical Investigation Brigham and Women's Hospital Boston Massachusetts USA; ^5^ Department of Medicine Jesse Brown VA Medical Center Chicago Illinois USA; ^6^ Northwestern University Feinberg School of Medicine Chicago Illinois USA; ^7^ University of Illinois Hospital and Health Sciences System Chicago Illinois USA; ^8^ Center for Business Models in Healthcare Chicago Illinois USA

**Keywords:** Black Veterans, distress screening, hospitalization, mortality, oncology, psycho‐oncology, supportive care services

## Abstract

**Background:**

We evaluated whether patients' initial screening symptoms were related to subsequent utilization of supportive care services and hospitalizations, and whether patient‐level demographics, symptoms, hospitalizations, and supportive care service utilization were associated with mortality in primarily low‐income, older, Black Veterans with cancer.

**Methods:**

This quality improvement project created collaborative clinics to conduct cancer distress screenings and refer to supportive care services at an urban, VA medical center. All patients completed a distress screen with follow‐up screening every 3 months. Supportive care utilization, hospitalization rates, and mortality were abstracted through medical records. Poisson regression models and cox proportional hazard models were utilized.

**Results:**

Five hundred and eighty five screened patients were older (*m* = 72), mostly Black 70% (*n* = 412), and had advanced cancer 54%. Fifty‐eight percent (*n* = 340) were screened only once with 81% (*n* = 470) receiving ≥1 supportive care service and 51.5% (*n* = 297) being hospitalized ≥1 time 18 months following initial screen. Symptom severity was significantly related to number of hospitalizations. Low mood was significantly related to higher supportive services (*p* < 0.001), but not hospitalizations (*p* ≥ 0.52). Pain, fatigue, physical function, nutrition, and physical symptoms were significantly associated with more supportive services and hospitalizations (*p* < 0.01). Twenty percent (*n* = 168) died; Veterans who were Black, had lower stage cancers, better physical health, and utilized less supportive care services had lower odds of mortality (*p* ≤ 0.01).

**Conclusion:**

Individuals with elevated distress needs and those reporting lower physical function utilized more supportive care services and had higher hospitalization rates. Lower physical function, greater supportive care use, higher stage cancer, and being non‐Black were associated with higher odds of death.

## 1 INTRODUCTION

Cancer diagnosis and treatment result in significant distress and supportive care needs for almost 50% of patients.^1^ Undertreated symptoms often result in worse patient outcomes including hospitalizations and mortality.^2^, ^3^ Veterans can be at increased risk for uncontrolled symptoms during their cancer trajectory due to having higher comorbidities (e.g., physical debility and post‐traumatic stress disorder).^4^, ^5^, ^6^, ^7^ Underrepresented minorities and older adults also face greater inequities having less access to medical care, lower health literacy, greater financial barriers, and higher rates of comorbid conditions.^4^, ^8^, ^9^, ^10^, ^11^ Identifying individuals at greatest risk for worse cancer outcomes remains relevant for patients, providers, health care systems, and payers alike and can help to illuminate intervention targets.^12^

In 2012, the National Comprehensive Cancer Network (NCCN) and American Society of Clinical Oncology (ASCO) established standards for distress screening and supportive care service referrals in oncology to address distress‐related needs and improve cancer outcomes.^13^, ^14^, ^15^, ^16^ Implementation and maintenance of distress screening programs vary in their make‐up, with most programs using modifications of existing protocols to match the unique needs of the population at hand, resulting in variable success.^1^, ^17^, ^18^, ^19^, ^20^ Studies that implemented distress screening and patient‐reported outcome programs demonstrate improved symptom detection,^4^ hospitalization prevention,^21^ and better connections to supportive care services,^3^, ^22^ and can result in increased referrals and utilization of outpatient supportive care services, and reduced hospitalizations.^23^, ^24^ Providing connections to supportive care services is important to help resolve distress during cancer; however, several barriers to successful supportive care visits have been identified (e.g. lack of insurance coverage).^12^, ^25^ Integrated interdisciplinary care models may be more effective at delivering supportive care services, particularly within closed health care systems with accessible supportive care services (e.g. Veterans Affairs [VA] Healthcare systems)^25^, ^26^; Yet, evaluation of such programs is lacking. Most evaluations of distress screening programs have been conducted in affluent, primarily White, NCI‐designated cancer centers, with fewer reports of screening programs and their outcomes in community clinics or those serving primarily underrepresented minorities.^9^, ^22^, ^24^

In response to the NCCN mandate, the Coleman Supportive Oncology Collaborative^1^ (CSOC), funded 25 Chicago‐area cancer care institutions to implement distress screening and develop agreed‐upon standards of care. The Jesse Brown VA Medical Center was chosen to evaluate cancer‐related concerns and improve access to supportive oncology. We previously published distress screening findings of our interdisciplinary, supportive care quality improvement (QI) initiative including feasibility of the primary project with screening rates, distress prevalence,^10^ and descriptions of the program's palliative care integration process within outpatient oncology^27^ in this mostly Black, older cohort of male Veterans. With the completion of the program's implementation cycles (2017–2020), we prospectively evaluated the associations between distress screening, subsequent supportive care utilization, hospitalization, and mortality in this underserved sample. We evaluated (1) whether patients' initial screening symptoms were related to subsequent utilization of supportive care services and hospitalizations and (2) whether patient‐level demographics, symptoms, hospitalizations, and supportive care service utilization were associated with mortality in this underserved population.

## 2 METHODS

### 2.1 Program, setting, and participants

The Jesse Brown VA serves 49,000 unique Veterans each year including predominately Black men of low socioeconomic status (SES) in Chicago, Cook County, IL. At Jesse Brown VA, patients receive care through four separate, half‐day general outpatient hematology‐oncology clinics, staffed by attendings and hematology/oncology fellows from two affiliated academic medical centers. In response to the NCCN/ACOS Commission on Cancer mandate 3.2 (now 5.2 in the 2020 standards),^28^ the grant was awarded to the Jesse Brown VA with a key emphasis on improving supportive oncology by increasing interdisciplinary team collaboration and supporting linkage across service lines through inter‐departmental supportive care referrals within the VA healthcare system. To do so, the project supported creation of a quality improvement team including a palliative care physician, palliative care advanced practice nurse, and psychology interns. The project supported integration of a QI team member within the outpatient general hematology‐oncology clinics to deliver screenings, evaluate patients' supportive care needs, and collaborate with oncology clinicians to refer patients to appropriate supportive care. Screening was first implemented within two clinics in the first 15 months of implementation and later expanded to a third clinic following additional staffing and after procedures were refined. In the participatory clinics, screening was offered to all Veterans with malignant cancer diagnoses and/or a new cancer diagnosis. The QI team member, prior to their assigned clinic, needed to predetermine and follow which patients qualified for screening as these were general hematology‐oncology clinics also serving patients with non‐malignant issues. In clinics with embedded QI team members, veterans could be flagged for an individual assessment either by the results of their completed screen or by referral from a medical oncology physician.^10^, ^27^

Ethical approval was received from the Institutional Review Board of the Jesse Brown VA as a QI Project (SQUIRE‐guidelines); not requiring written informed consent (GCL#21270).^29^ This project was implemented between January 2017–January 2020. Methods for complete program development and initial findings are reported elsewhere.^10^

### 2.2 Screening instrument and clinical procedures

Veterans receiving care for a new diagnosis and/or oncology‐related needs at the Jesse Brown VA completed a distress screening during program initiation. All veterans completed screening, unless unable to read or unwilling to do so. Follow‐up screening occurred at 3‐month intervals. Veterans completed paper‐form screening as they awaited being seen by their oncologists, and returned the form to the triage nurse, who then shared them with the patients' treating oncologist. The QI team reviewed the screening results for elevated symptoms (previously identified cut‐offs; see measure references respectively). Oncology fellows were trained by the QI team before program initiation to discuss relevant needs with the patient, or to conduct a “warm hand‐off” to the QI team to complete the clinical evaluation. Appropriate referrals for supportive care services were then placed by either the oncology fellow or QI team. To assist with referral initiation, the QI team created and distributed a referral action guide for internal supportive oncology referrals in each clinic room. Supportive care services identified as relevant to oncology needs included outpatient palliative care, social work, pain management, mental health, chaplaincy, and nutrition services. Distress screening and treatment integration occurred within one physical space with co‐located QI team members functioning in unison with oncology staff (physicians, nurses, and administrative staff), allowing real‐time discussion between oncology staff (attendings, fellows, nurses) and the QI team members to review clinical evaluations and subsequent referrals to address patients' needs. Previous analyses identified that 25–50% of all new consults and similar rates of current patients were screened^10^, ^27^; Patients were not screened because they either had primary hematologic concerns, were not receiving their primary treatment through the VA, denied screening often because they denied symptoms/needs, or because they were missed during an appointment time. For patients with elevated psychosocial needs, 36% were connected with psychosocial services before screening and 77% were connected with psychosocial care after screening.^10^

The study utilized the “Patient Screening Questions for Supportive Care” developed by an interdisciplinary group of 35 organizations through the CSOC.^30^ This measure includes a compilation of validated measures combined for this QI program (10 min to complete).^10^ The screening questionnaire included: the NCCN Guidelines Distress Management Needs Assessment Problem List‐ Physical and Other Concerns (number of “physical symptoms”; 20 total e.g., breathing, constipation, fevers, skin issues, substance use),^31^ and Practical Concerns (e.g., issues paying for food, housing, transportation, insurance coverage), the Patient‐Reported Outcomes Measurement Information System (PROMIS)^32^: Pain Intensity (3a, 10‐point Likert scale), Fatigue‐SF (4‐item; 5‐point Likert scale), and Physical Function (5‐item; e.g. go up and down stairs, able to get out of bed; 5‐point Likert scale) subscales, the Mini Nutrition Assessment short‐form (MNA‐SF; binary responses yes/no; concerns for weight loss/gain and issues with taste and food),^33^ and the Patient Health Questionnaire (PHQ)‐4 (symptoms of anxiety and depression; 4‐point Likert scale; scoring: 0–4 = none/minimal, 5–9 = mild, 10–14 = moderate, 15–19 = moderately severe, 20–27 severe).^34^ For all measures, higher scores represent higher rates of that symptom, except physical function where higher scores indicate worse physical function.

Relevant data were extracted from the medical record with a censorship date of 18 months from initial screen. A trained research assistant (F.S.) abstracted demographic data, mortality status and date, hospitalizations, and supportive care service utilization. Due to medical record limitations as the system does not track this data, we were unable to evaluate whether each service visit was directly related to a distress screen referral; however, we only abstracted supportive care services that occurred after the patients' initial screening date. Our previous analysis identified that very few patients received supportive care services at the start of this program.^10^

### 2.3 Statistical analysis

Patients with cancer who had completed at least one distress screen were included in analyses. We used two separate univariate Poisson regression models with robust standard errors to evaluate the association of symptom severity at screen 1 for symptoms (independent variables) of (1) pain, (2) depression, and (3) fatigue, (4) levels of physical function, (5) rates of physical symptoms, (6) nutrition concerns, and (7) practical concerns with the dependent variables: number of supportive care services and number of hospitalizations; For practical concerns, the variable responses had 48% zero values and limited variability above 0, a binary logistic regression model (i.e., the response was 0 vs. >0) was utilized instead.

Cox proportional multivariable hazard model was utilized to evaluate the association of age, race, cancer stage, list of symptoms, number of hospitalizations, and number of supportive care services with time to death (dependent variable). Variables including age, number of hospitalizations, symptoms of pain, depression, and fatigue, rates of physical symptoms, practical concerns, and nutritional concerns were removed from the multivariable analysis because Pearson's correlations were greater than 0.90 with other model variables. Analyses were performed in SAS 9.3 (SAS Institute).

## 3 RESULTS

In total, 585 patients were successfully screened once or more for distress during their outpatient oncology care (1 screen, *n* = 585; 2 screens [42.2%]; 3 screens [18.1%]; 4 screens [8.3%]; 5 screens [2.4%]; 6 screens [1.5%]). Patients were 72 years old on average (SD = 9.5), mostly male (96%), Black (70%), had advanced‐stage cancer (for all solid organ tumors, except prostate, stage III/IV = 53.6%); with 213 missing or un‐staged hematologic or prostate cancers, and reported mild depressive/anxiety symptoms (PHQ‐4 *m* = 2.49 [SD = 3.165]). At initial screening, patients reported elevated symptoms of pain, fatigue, and physical function [Pain *m* = 57.3, SD = 1.9 (t‐score), Fatigue *m* = 50.8, SD = 2.5 (T‐score), Physical Function m = 19.0, SD = 5.5(T‐score)]. More than half of the patients reported elevated rates of physical concerns [≥4 = 61%], 52% reported practical concerns for paying for food, housing, transportation, or work‐related needs, and 54% reported nutrition concerns. Evidence of the program's success, most patients were seen by one or more supportive care services in the 18 months following their initial distress screen (81.2%). About half of the sample was hospitalized at least once in the 18 months following their initial distress screen (52.8%), with 29.4% being hospitalized at least 2 or more times, and a quarter of the sample died within 18 months of their initial screen (26.8%). See Table 1.

**TABLE 1:** Patient demographics who completed distress screen during outpatient oncology visits (*n* = 585)

Variable	*n* (%)	M (SD)
Age		71.78 (9.50)
Stage		
I	54 (14.6)	
II	45 (12.2)	
III	74 (20)	
IV	197 (53.6)	
Unstaged/unknown	215 (36.7)	
Cancer type		
Lung	125 (21.6)	
Prostate	97 (16.8)	
Breast	56 (9.7)	
Multiple Myeloma	47 (8.1)	
ENT	33 (5.7)	
Lymphoma	33 (5.7)	
Leukemia	29 (5.0)	
Colorectal	27 (4.7)	
Gastric	24 (4.2)	
Renal	19 (3.3)	
Pancreatic	17 (2.9)	
Urinary	12 (2.1)	
Hepatocellular	11 (1.9)	
Other	44 (7.6)	
Race		
Black	412 (70.3)	
White	137 (23.4)	
Other	9 (1.6)	
Number of hospitalizations		
0	281 (48.6)	
1	127 (22.0)	
2	74 (12.8)	
3	45 (7.8)	
4	16 (2.8)	
≥5	35 (6.1)	
Number of supportive care services utilized in18 months following screening		
None	109 (18.8)	
1	108 (18.7)	
2	79 (13.6)	
3	64 (11.1)	
4	45 (7.8)	
5	56 (9.7)	
6	73 (12.6)	
≥7	45 (7.8)	
Rates of supportive care services		
Palliative care	158 (27.3)	
Nutrition	307 (53.0)	
Social work	363 (62.7)	
Psychology	72 (12.5)	
Psychiatry	119 (20.6)	
Physical therapy	255 (44.0)	
Occupational therapy	195 (33.7)	
Pain clinic	27 (4.7)	
Chaplaincy	185 (32.0)	
Mortality w/in 18 months from screening	168 (26.8)	

### 3.1 Association of initial symptom severity with no. of subsequent supportive care services and hospitalizations

Symptoms of depression/anxiety (9% increase, *p* ≤ 0.001), pain (5% increase, *p ≤* 0.0001), fatigue (5% increase*, p ≤* 0.001), physical symptoms (7% increase, *p* ≤ 0.0001), and nutritional concerns (11% increase, *p* ≤ 0.0001) during their initial screen were associated with significant increases in the number of utilized supportive care services. For individuals with improved physical function, this was associated with a 4% decrease (*p* ≤ 0.0001) in the number of supportive care services across participants (Table 2A). Similarly, for individuals with increased symptoms of pain (3% increase, *p* = 0.013) and fatigue (3% increase, *p* = 0.002), higher numbers of physical symptoms (4% increase, *p* = 0.017) and nutrition concerns (6% increase, *p* = 0.015) was significantly associated with more hospitalizations, but symptoms of anxiety/depression and practical concerns were not associated with significantly greater hospitalizations. For individuals with improved physical function, this was associated with a 3% decrease (*p* ≤ 0.0001) in the number of hospitalizations across the sample (Table 2B).

**TABLE 2:** The association of symptom severity with number of supportive care services and number of hospitalizations using poisson regression models

A. The association of symptom severity with number of supportive care services				
	Estimate	Robust standard error	*p*	Incident rate ratios (IRR)
Depression/anxiety	0.0826	0.0222	≤ 0.001	1.09 (1.04, 1.13)
Pain	0.0491	0.0091	≤ 0.0001	1.05 (1.03, 1.07)
Fatigue	0.0521	0.0083	≤ 0.001	1.05 (1.04, 1.07)
Physical function	−0.0419	0.0051	≤ 0.0001	0.96 (0.95, 0.97)
Physical symptoms	0.0698	0.0129	≤ 0.0001	1.07 (1.05, 1.10)
Nutrition concerns	0.1088	0.0210	≤ 0.0001	1.11 (1.07, 1.16)

	Odds ratio estimate	95% CI	*p*	
Practical concerns	1.047	(0.977, 1.122)	0.19	

B. The association of symptom severity with number of hospitalizations				
	Estimate	Robust standard error	*p*	Incident rate ratios (IRR)
Depression/anxiety	0.0187	0.0288	0.52	1.02 (0.96, 1.08)
Pain	0.0325	0.0130	0.013	1.03 (1.01, 1.06)
Fatigue	0.0289	0.0091	0.002	1.03 (1.01, 1.05)
Physical function	−0.0327	0.0084	≤ 0.0001	0.97 (0.95, 0.98)
Physical symptoms	0.0362	0.0152	0.017	1.04 (1.01, 1.07)
Nutrition concerns	0.0564	0.0231	0.015	1.06 (1.01, 1.11)

	Odds ratio estimate	95% CI	*p*	
Practical concerns	0.995	(0.913, 1.085)	0.91	

*Note*: *p‐value calculated from maximum likelihood estimates. PHQ‐4: depressive/anxiety symptoms. Depression and anxiety: PHQ4. Physical function: PROMIS Physical function. Physical symptoms: NCCN checklist. Nutrition concerns: MNA‐SF.

### 3.2 Associations with mortality

In univariable analyses, patients who were older or White, had stage 4 cancer, had higher utilization of supportive care services, increased hospitalizations, and self‐reported higher symptoms of depression/anxiety, pain, and fatigue, lower physical function, increased physical symptoms, practical concerns, and nutrition concerns had significantly higher odds of mortality at 95% confidence limit (*p* ≤ 0.05, Table 3). After removing the variables with elevated multicollinearity in the multivariable model (See Appendix A), we found that compared to patients with stage 0 or 1 cancers, the risk of death was 3 times higher [Hazard Ratio (HR): 3.0, CI:1.43–6.33, *p* = 0.004] for patients with stage 4 cancers (Table 4). Compared to non‐Black participants, the risk of death *decreased* by 38% (HR:0.62, CI:0.39–0.98, *p* = 0.041) for Black patients. For each increase in utilized supportive care services, the risk of death *increased* by 11% (HR:1.11, CI:1.01–1.23, *p* = 0.028). For each one‐unit increase in Physical Function, the risk of death decreased by 9% (HR: 0.91, CI:0.87–0.94, *p* < 0.001).

**TABLE 3:** The associations of age, list of symptoms, number of hospitalizations and number of supplemental services with time to death using univariable Cox proportional hazards regression models

Parameter	Total	No. of events	Estimate	Standard error	*p*‐value	Hazard ratio (HR)
Age	574	164	0.0141	0.00834	0.093	1.01 (0.99–1.03)
No. of supportive care services	574	164	0.2482	0.0337	≤ 0.0001	1.28 (1.20–1.37)
No. of hospitalization	573	164	0.0865	0.0317	0.006	1.09 (1.03–1.16)
Depression/anxiety	530	145	0.0874	0.0240	≤ 0.0.001	1.09 (1.04–1.14)
Pain	427	130	0.0636	0.0245	0.009	1.07 (1.02–1.12)
Fatigue	535	148	0.0958	0.0168	≤0.0001	1.10 (1.07–1.14)
Physical function	523	146	−0.1133	0.0146	≤0.0001	0.89 (0.87–0.92)
Physical symptoms	580	164	0.0591	0.0195	0.002	1.06 (1.02–1.10)
Practical concerns	580	164	−0.0734	0.0658	0.26	0.93 (0.82–1.06)
Nutritional concerns	580	164	0.0868	0.0256	≤0.0.001	1.09 (1.04–1.15)
Stage						
2	369	114	0.38301	0.48592	0.4306	1.47 (0.57–3.80)
3	369	114	0.23742	0.44936	0.5973	1.27 (0.53–3.06)
4	369	114	1.33484	0.37038	0.0003	3.80 (1.84–7.85)
0 or 1	Ref.	Ref.	Ref.	Ref.	Ref.	Ref.
Race						
Black	554	152	−0.1941	0.1779	0.2755	0.82 (0.58–1.17)
Non‐Black	Ref.	Ref.	Ref.	Ref.	Ref.	Ref.

*Note*: *Depression and anxiety: PHQ4. Physical function: PROMIS Physical function. Physical symptoms: NCCN checklist. Nutrition concerns: MNA‐SF.

**TABLE 4:** The association of cancer stage, race, symptoms, no. of supportive care services with time to death using a multivariable Cox proportional hazards regression model

Parameter	Parameter estimate	Standard error	Chi‐Square	*p*	Hazard ratio	95% Hazard ratio confidence limits	
Physical function	−0.09847	0.01942	25.7191	<0.0001	0.906	0.872	0.941
Supportive care service utilization	0.10760	0.04889	4.8440	0.0277	1.114	1.012	1.226
Stage							
Stage 0/1	Ref.	Ref.	Ref.	Ref.	Ref.	Ref.	Ref.
Stage 2	0.22504	0.50324	0.2000	0.6547	1.252	0.467	3.358
Stage 3	−0.10056	0.48818	0.0424	0.8368	0.904	0.347	2.354
Stage 4	1.10155	0.37904	8.4459	0.0037	3.009	1.431	6.325
Race							
Black	−0.48157	0.23585	4.1691	0.0412	0.618	0.389	0.981
Non‐Black	Ref.	Ref.	Ref.	Ref.	Ref.	Ref.	Ref.

*Note*: *Variables including age, number of hospitalizations, PHQ4, PROMIS Pain, PROMIS Fatigue, Physical Symptoms, number of practical concerns, nutritional concerns were removed from the multivariable analysis as they surpassed multicollinearity cutoffs, Pearson's correlation ≥0.90 with other variables, see Appendix A. Physical function: PROMIS Physical function.

## 4 DISCUSSION

The results of this prospective, longitudinal QI project in a sample of 585 primarily older, Black, male Veterans at an urban VA medical Center, identified that patient‐reported factors and unmet needs were significantly associated with higher rates of subsequent supportive care service utilization, hospitalizations, and mortality. Within 18 months following an initial distress screen, 81% of patients were seen by one or more supportive care services, 52% had been hospitalized at least once, and 27% had died. Elevated patient‐reported needs during initial distress screening were significantly associated with the number of supportive care services utilized and number of hospitalizations. Notably, patient‐reported symptoms including psychological symptoms were related to mortality on univariable analysis, yet in multivariable models, individuals who were Black and those who were more physically fit had lower odds of death, and those with worse stage cancers or who utilized more supportive care services had higher odds of death.

Our previous paper outlining distress rates confirmed that this study sample reported higher rates of undertreated needs in this VA sample compared to general U.S. samples.^10^ While our findings parallel previous observations that increased distress and undertreated physical and practical needs were related to increased supportive care service utilization,^3^, ^35^ this study identified that in a sample of mostly male Veterans who face distinctive challenges given their prolonged exposure to trauma, chronic health comorbidities, and elevated financial needs, individuals utilize services at *higher rates* compared to other samples.^8^, ^35^, ^36^ Encouragingly, these findings provide further validity for the association between distress screening and subsequent supportive care service utilization. Findings also provide distinct rates of supportive care service utilization following screening, expanding on Van Ryn et al.'s 2014 national survey of Veterans that identified non‐White patients were more likely to receive the help that they wanted during their cancer care compared to their white counterparts.^4^ Our findings also support previous theories that the Veteran's Health Administration's focus on reducing racial disparities in care may be succeeding.^4^, ^37^ It may also be the case that individuals were more likely to engage in supportive care services because these services are often covered within VA care and do not result in substantially increased costs; this difference may be especially noticeable as rates of financial toxicities from cancer continue to increase.^9^, ^38^ The results highlight that integration of distress screening through interdisciplinary care coordination served to identify elevated symptoms which were associated with increased supportive care service utilization in this group of mostly older, Black, male Veterans with cancer.

Multivariable analysis identified that odds of mortality were lower for Black patients compared to their non‐Black counterparts, in addition to those with lower‐stage cancers, higher physical function, and lower supportive care service utilization. While this disparity in outcomes based on race seems surprising given the overall increased mortality for Black individuals,^39^, ^40^ some evidence suggests that rates of death from cancer for Black individuals are decreasing.^41^ Specifically, studies of prostate cancer and lung cancer outcomes, the two most prominent cancers in this sample, identified that Black patients had a lower risk of death compared to whites.^42^, ^43^, ^44^ Yet, stage of cancer, SES including educational attainment, health co‐morbidities, and other sociodemographics may be more predictive of mortality than race, therefore confounding the results of this analysis.^45^, ^46^, ^47^ Contrary to previous findings, supportive care service utilization, while high, was also related to increased mortality.^48^ It may be that Veterans in this sample who used supportive care services were more ill and older, especially compared to other samples and evidenced by a large proportion having advanced‐stage disease and high rates of mortality within 18 months of screening, where supportive care services may have less of a clinical impact on mortality.^49^ All supportive care services are also not created equal, and due to the lack of statistical power, we were unable to evaluate the relative impact of each service on mortality. Future research is required to understand how the types and variety of input from different supportive care services could be mediating any subsequent changes in distress outcomes. The mean age of this cohort was 72 years old, and there existed a consistent significant relationship between those who were more physically active having lower rates of supportive care service utilization, hospitalizations, and mortality. These findings support the strong relationship between age, physical function, and relevant cancer outcomes.

To our knowledge, few studies evaluating interdisciplinary distress screening programs and related healthcare utilization and mortality exist among Veterans. This study has several limitations. No comparison group existed to evaluate differences between those who engaged in the screening program and those did not, or reasons for lack of screening, and we did not control for clinic differences in our analyses. Future studies comparing outcomes between groups may identify particular benefits of screening. Additionally, given limitations in the medical record, we were unable to confirm that supportive care service utilization was a direct result of screening and referral from the program. However, few patients prior to distress screening were receiving supportive care services. Findings of this study may be generalizable only to similar samples of male veterans from underserved communities; few individuals were female (around 4%) and many cancers were unstaged (e.g., prostate and blood). Female veterans face unique challenges that may vary from their male counterparts, particularly within the cancer context, and should therefore be evaluated in future studies. Lastly, findings from VA hospitals may not be generalizable to non‐VA centers; however, previous comparisons between VA and fee‐for‐service Medicare services indicated that VA cancer care may be either equal or better than Medicare counterparts.^50^

In conclusion, care models that are integrated within oncology to identify and treat supportive care needs can successfully result in increased care integration that likely improves quality of life and reduces cancer burden for underserved patients and health care systems alike. Individuals with elevated distress needs and who report lower physical function will utilize more supportive care services and have higher rates of hospitalizations during cancer; those who are more active, Black, have lower stage cancer, and use less supportive care services may have lower odds of mortality.

## AUTHOR CONTRIBUTIONS

**Matthew J. Allsop:** Methodology (equal); writing – original draft (supporting); writing – review and editing (supporting). **Subrina Farah:** Formal analysis (lead); methodology (supporting); writing – review and editing (equal). **Farah Salim:** Data curation (equal); project administration (supporting); writing – review and editing (supporting). **Joshua Hauser:** Conceptualization (supporting); funding acquisition (equal); project administration (supporting); resources (lead); writing – review and editing (supporting). **Ashton R. Baltazar:** Writing – review and editing (supporting). **Robert E Molokie:** Conceptualization (supporting); data curation (supporting); project administration (supporting); resources (supporting); writing – review and editing (supporting). **Jane Weber:** Data curation (supporting); resources (supporting); writing – review and editing (supporting). **Christine Brezina Weldon:** Conceptualization (equal); methodology (supporting); project administration (equal); writing – review and editing (supporting). **Lawrence Feldman:** Data curation (supporting); project administration (supporting); writing – review and editing (supporting). **Joanna Martin:** Conceptualization (equal); data curation (equal); funding acquisition (lead); project administration (equal); resources (equal); writing – original draft (supporting); writing – review and editing (supporting).

## FUNDING INFORMATION

Coleman Foundation (JM, DRA, JH, LF), National Palliative Care Research Center (DRA).

## CONFLICT OF INTEREST

The authors have no disclosures to report.

## Supporting information


Appendix A.

## Data Availability

The data that support the findings of this study are available on request from the corresponding author. The data are not publicly available due to privacy or ethical restrictions.
