# Correction to “Oncology distress screening within predominately Black Veterans: Outcomes on supportive care utilization, hospitalizations, and mortality”

**DOI:** 10.1002/cam4.6366

**Published:** 2023-07-28

Azizoddin DR, Allsop M, Farah S, et al. Oncology distress screening within predominately Black Veterans: Outcomes on supportive care utilization, hospitalizations, and mortality. *Cancer Med*. 2023;12:8629‐8638. https://doi.org/10.1002/cam4.5560

In the originally published article, the following VA attribution and disclaimer were missing from the Acknowledgments section.

“This material is the result of work supported with resources and the use of facilities at the Jesse Brown VA Medical Center, Chicago, Illinois.”

“The views expressed in this article are those of the authors and do not necessarily reflect the position or policy of the Department of Veterans Affairs or the United States government.”

We apologize for this error.

