# Supplementary material for: Oncology distress screening within predominately Black Veterans: Outcomes on supportive care utilization, hospitalizations, and mortality
Source: Cancer Med. 2022 Dec 27;12(7):8629–38. doi: 10.1002/cam4.5560 (PMC10134375; doi:10.1002/cam4.5560)
Supplement: Supplementary file 1 — Appendix A. [file CAM4-12-8629-s001.docx]

Appendix A. Correlation Matrix of Mortality Model Variables

|  | Number of hospitalizations | PHQ4 | PROMIS Pain | PROMIS Fatigue | Physical Symptoms | Number of practical concerns | Nutritional concerns |
| --- | --- | --- | --- | --- | --- | --- | --- |
| PROMIS Physical Function | 0.87 | 0.76 | 0.98 | 0.86 | 0.78 | 0.89 | 0.81 |
| Supportive Care Services | 0.93 | 0.88 | 0.82 | 0.93 | 0.76 | 0.80 | 0.79 |
| Stage | 0.8 | 0.77 | 0.78 | 0.92 | 0.81 | 0.84 | 0.85 |
| Race | 0.96 | 0.86 | 0.78 | 0.81 | 0.77 | 0.8 | 0.73 |
